# Polarized Cytokine Release Triggered by P2X7 Receptor from Retinal Pigmented Epithelial Cells Dependent on Calcium Influx

**DOI:** 10.3390/cells9122537

**Published:** 2020-11-24

**Authors:** Xiaolei Shao, Sonia Guha, Wennan Lu, Keith E. Campagno, Jonathan M. Beckel, Jason A. Mills, Wenli Yang, Claire H. Mitchell

**Affiliations:** 1 Shenzhen Eye Hospital, School of Optometry, Shenzhen University, Shenzhen 518060, China; sxl320@hotmail.com; 2 Department of Anatomy and Cell Biology, University of Pennsylvania, Philadelphia, PA 19104, USA; guha@jsei.ucla.edu (S.G.); JMBECKEL@pitt.edu (J.M.B.); 3 Jules Stein Eye Institute, University of California Los Angeles, Los Angeles, CA 90095, USA; 4 Department of Basic and Translational Science, University of Pennsylvania, Philadelphia, PA 19104, USA; wennan@upenn.edu (W.L.); campagno@pennmedicine.upenn.edu (K.E.C.); 5 Department of Pharmacology and Chemical Biology, University of Pittsburgh, Pittsburgh, PA 15261, USA; 6 Department of Ophthalmology, University of Pennsylvania, Philadelphia, PA 19104, USA; jamills44@gmail.com; 7 Kirby Center for Molecular Ophthalmology and Center for Advanced Retinal and Ocular Therapeutics (CAROT), University of Pennsylvania, Philadelphia, PA 19104, USA; 8 Department of Medicine and Institute for Regenerative Medicine, University of Pennsylvania, Philadelphia, PA 19104, USA; wenliyan@pennmedicine.upenn.edu; 9 Department of Physiology, University of Pennsylvania, Philadelphia, PA 19104, USA

**Keywords:** P2X7, retina, IL-6, IL-1β, cytokine release, innate immunity, calcium-dependent vesicular release, inflammation

## Abstract

Cytokine release from non-inflammatory cells is a key step in innate immunity, and agonists triggering cytokine release are central in coordinating responses. P2X7 receptor (P2X7R) stimulation by extracellular ATP is best known to active the NLRP3 inflammasome and release IL-1β, but stimulation also leads to release of other cytokines. As cytokine signaling by retinal pigmented epithelial (RPE) cells is implicated in retinal neurodegeneration, the role of P2X7R in release of cytokine IL-6 from RPE cells was investigated. P2X7R stimulation triggered IL-6 release from primary mouse RPE, human iPS-RPE and human ARPE-19 cells. IL-6 release was polarized, with predominant rise across apical membranes. IL-6 release was inhibited by P2X7R antagonists A438079, A839977, and AZ10606120, but not the NRTI lamivudine (3TC), P2X1R antagonist NF279, or P2Y1R antagonist MRS2179. P2X7R-mediated IL-6 release required extracellular Ca^2+^ and was blocked by Ca^2+^ chelator BAPTA. IL-6 release and Ca^2+^ elevation occurred rapidly, consistent with vesicular IL-6 staining in unstimulated cells. P2X7R stimulation did not trigger IL-1β release in these unprimed cells. P2X7R-mediated IL-6 release was enhanced in RPE cells from the ABCA4^−/−^ mouse model of retinal degeneration. In summary, P2X7R stimulation triggers rapid Ca^2+^-dependent IL-6 release across the apical membrane of RPE cells.

## 1. Introduction

Release of cytokines from non-immune cells is a key step in innate immune responses, and the receptors that activate this cytokine release represent critical targets in controlling the response [1,2]. Stimulation of the P2X7 receptor for extracellular ATP is associated with the assembly of the NOD-, LRR- and pyrin domain-containing protein 3 (NLRP3) inflammasome and the resulting release of cytokines interleukin (IL)-1β and IL-18 [3,4]. As IL-1β is considered a “master cytokine”, capable of increasing expression and release of other cytokines [5], stimulation of the P2X7 receptor can be important in coordinating components of the innate immune response. Whether the P2X7 receptor can also trigger cytokine upregulation and release independent of IL-1β is less well studied. This is relevant as the NLRP3 inflammasome requires “priming” to increase expression of key components, such as NLRP3 itself, before the activation stage can occur [6], and priming of inflammasome components may be rate-limiting in chronic age-dependent diseases [7]. The involvement of the P2X7 receptor in release of cytokines other than IL-1β may thus have implications for earlier stages in chronic inflammatory diseases.

Retinal pigmented epithelial (RPE) cells form the blood–retinal barrier in the outer retina and provide numerous supportive functions for the adjacent photoreceptors [8,9]. The innate immune responses of RPE cells have been implicated in both protective and detrimental roles in ocular pathologies [10]. While the release of inflammasome products IL-1β and IL-18 from RPE cells has been studied in several models of retinal stress [11,12,13], NLRP3 in human tissue was detected only in RPE cells adjacent to regions undergoing the terminal geographic atrophy phase of age-dependent macular degeneration [14]. This suggests that priming of NLRP3 and other components represented a late stage development, and that the cytokine response from unprimed cells may be of greater relevance in the early stages of disease.

IL-6 signaling is complex; although traditionally thought of as a pro-inflammatory cytokine, IL-6 can also have protective effects, depending on levels of the soluble IL-6 receptor [15]. Recently, IL-6 expression was reported to be elevated in RPE cells and stroma of patients with exudative age-related macular degeneration (AMD), and downregulation of IL-6 reduced subretinal fibrosis in mice models [16]. Recent work also implicated the P2X7 receptor in the release and upregulation of IL-6 in optic nerve head astrocytes and retinal ganglion cells [17].

The current study examines whether stimulation of the P2X7 receptor can trigger release of cytokine IL-6 from unprimed RPE cells. The findings suggest that the P2X7 receptor can trigger a rapid release of IL-6 from RPE cells that is dependent on increased intracellular Ca^2+^ and not reliant on IL-1β.

## 2. Materials and Methods

### 2.1. Isolation and Culture of Mouse RPE Cells

All mice were treated in accordance with University of Pennsylvania Institutional Animal Care and Use Committee (IACUC #804588). Mice were reared at 5–15 lux and sacrificed with a CO_2_ overdose. Mouse eyes were isolated and processed as described previously [18]. In brief, after enucleation, intact eyes were incubated in 2% dispase and 0.4 mg/mL collagenase IV for 45 min. Eyes were then incubated for 20 min in growth medium containing Dulbecco’s Modified Eagle Medium (DMEM) and Ham’s F12 medium with 3 mM L-Glutamine, 100 µg/mL streptomycin, and 2.5 mg/mL Fungizone and/or 50 µg/mL gentamicin and 10% fetal bovine serum (FBS, all Thermo Fisher, Inc., Waltham, MA, USA). Globes were separated at the ora serata, the retina removed and sheets of RPE cells were dissected away from the choroid with fine forceps. These RPE sheets were collected in growth media and triturated to form single cell suspensions. Mouse RPE cells were seeded onto surfaces coated with 0.05% poly-L-lysine and 10 μg/mL laminin. ABCA4^−/−^ mice were a kind gift from Dr. Gabriel Travis of the Jules Stein Eye Institute, UCLA. All materials were purchased from Sigma Chemical Corp (St. Louis, MO, USA) unless otherwise indicated.

### 2.2. Human Induced Pluripotent Stem (iPS) Cell Culture and Differentiation into Induced RPE Cells

Undifferentiated iPS cells (Control 1 (CHOPWT10). Control 2 (PENN123i-SV20)), and Control 3 (PENN067i-312-1) were derived and characterized as previously published [19,20]. The University of Pennsylvania human subjects research Institutional Review Board (IRB #814132) approved the collection of samples and all subjects gave written informed consent. Induced pluripotent stem cells were maintained in pluripotent stem cell (PSC) medium (DMEM/F12 (50:50), 1× Glutamax, 1× penicillin/streptomycin, 15% knockout serum replacement, 1x non-essential amino acids, 0.1 mM β-mercaptoethanol, and 10 ng/mL of basic fibroblast growth factor on 0.1% gelatin coated dishes (all Thermo Fisher, Inc.). Following the protocol Duong et al. [21], feeder-free differentiation conditions, small molecules, and growth factors were used to induce the generation of RPE cells. At day 35 of differentiation, cells were harvested using accutase and passaged twice in 1% growth factor reduced Matrigel coated dishes in X-Vivo 10 media plus 2 μM of thiazovivin for one day, followed by X-Vivo 10 media alone. Cells were also grown on 12-well Transwell inserts with 0.4 µm pore size (Corning Inc, Corning, NY, USA). An EVOM2 system (World Precision Instruments, Sarasota, FL, USA) with a STX2 probe was used to measure total transepithelial electrical resistance (TEER) once a week. All TEER values were normalized to the area of the membrane (1.44 cm^2^) and corrected for the resistance without cells.

### 2.3. ARPE-19 Cells

ARPE-19 cells (ATCC, Manassas, VA, USA) were grown to confluence in 25 cm^2^ primary culture flasks in a 1:1 mixture of DMEM/F12 with 3 mM L-Glutamine, 100 µg/mL streptomycin, 100 U/mL penicillin, and 10% FBS (all Thermo Fisher, Inc.) as described [22]. The concentration of FBS was reduced to 1% upon confluence to encourage differentiation. Cells were grown on standard tissue culture plates or Transwell inserts using the methods described above.

### 2.4. Cytokine Measurement

After washing, cells were incubated with P2X7 receptor agonist 2′(3′)-O-(4-Benzoylbenzoyl)adenosine-5′-triphosphate (BzATP) and supernatant was collected at the indicated times. IL-6 levels were determined using the QuantiGlo IL-6 Immunoassay or Quantikine enzyme-linked immunosorbent assay (ELISA) kits (both R&D Systems, Minneapolis, MN, USA) with signal detected using a luminometer (Thermo Fisher, Inc.) or SpectraMax Absorbance reader (Molecular Devices, San Jose, CA, USA), respectively. Parallel approaches were used to detect IL-1*β* levels using the QuantiGlo Human IL-1B/IL-1F2 Immunoassay (R&D Systems). Mg^2+^-free isotonic solution [(in mM) 105 NaCl, 5 KCl, 6 HEPES acid, 4 Na HEPES, 5 NaHCO_3_, 60 mannitol, 5 glucose, and 1.3 CaCl_2_] was used in some experiments as Mg^2+^ is reported to block the P2X7 receptor [23]. The absolute levels of IL-6 varied across experiments, perhaps due to small differences in extracellular volume or the freshness of the IL-6 solution used for standard curves.

### 2.5. P2X7, P2X1 and P2Y1 Antagonists

Cells were washed with Mg^2+^-free isotonic solution then preincubated with antagonists for 15 min at 37 °C. Solution was replaced with one containing isotonic control solution or BzATP ± antagonist. Cells were incubated for 30 min at 37 °C, after which supernatant was collected. IL-6 levels were determined with using a Quantikine ELISA kit as described above. Antagonists used were P2X7 antagonists A438079, AZ10606120, A839977, P2X1 antagonist NF 279, P2Y1 antagonist MRS2176, and lamivudine (3TC). All antagonists were purchased from Tocris/Biochine Corp. (Minneapolis, MN, USA) except for lamivudine (Toronto Research Chemicals, Toronto, ON, Canada).

### 2.6. Calcium Measurements

Intracellular Ca^2+^ was measured based on methods previously described in detail [24]. In brief, ARPE-19 cells were grown and loaded with 5 μM Ca^2+^ indicator dye Fura-2 AM and 2% pluronic F127 (Thermo Fisher, Inc.). Coverslips were mounted in a perfusion chamber and cells visualized using a ×40 objective on a Nikon Diaphot microscope (Nikon USA, Melville, NY, USA). The fura-2 dye in cells was alternatively excited at 340 and 380 nm, and the fluorescence emitted >520 nm was imaged with a charge-coupled device camera and analyzed (all Photon Technologies International, Lawrenceville, NJ, USA). Calibration was performed using ionomycin in the presence of high Ca^2+^ and Ca^2+^ free solutions at pH 8.0.

### 2.7. Immunocytochemistry

ARPE-19 cells were fixed with 4% paraformaldehyde in 100 mM phosphate buffered saline (PBS) for 15 min. After washing, cells were permeabilized and autofluorescence quenched in PBS containing 20 mM glycine, 75 mM ammonium chloride and 0.1% Triton X-100 for 8 min, followed by a 2 min incubation with this solution plus 0.5% sodium dodecyl sulfate. Cells were blocked in 0.25% fish skin gelatin, 0.025% saponin and 10% donkey serum in PBS for 1 h at 25 °C, followed by 5 min in high salt PBS. Cells were incubated overnight in a rabbit primary polyclonal antibody against IL-6 (Abcam, Cambridge, MA, USA; #ab6672, 1:500 in blocking solution) at 4 °C. After washing, cells were incubated for 1 h donkey anti-rabbit Alexa 488 (Abcam, 1:1000, in blocking solution) and F-actin counterstain (Alexa-594 phalloidin, 1:1000, Thermo Fisher Inc.) at 25 °C. Nuclei were counterstained with 4′,6-diamidino-2-phenylindole (DAPI; 1:10,000, 1 min). Antibody-antigen binding was fixed for 15 min in 4% paraformaldehyde. Staining was visualized using a Nikon Eclipse E600 epifluorescence microscope, and separate channels combined using Adobe Photoshop (San Jose, CA, USA) according to accepted protocols.

### 2.8. Data Analysis

All data are expressed as mean ± standard error of the mean. Significance was defined as *p* < 0.05 and was determined using a student’s *t*-test or one-way Analysis of Variance (ANOVA) with the F value (degrees of freedom for numerator/denominator), followed by appropriate Post-hoc test. Data were analyzed using SigmaStat software (Systat Software, Inc., San Jose, CA, USA) or GraphPad Prism version 8.0.0 for Windows (GraphPad Software, San Diego, CA, USA, www.graphpad.com).

## 3. Results

### 3.1. IL-6 Release Triggered by P2X7 Receptor Activation in Mouse, iPS-RPE and ARPE-19 Cells

**Figure 1:**
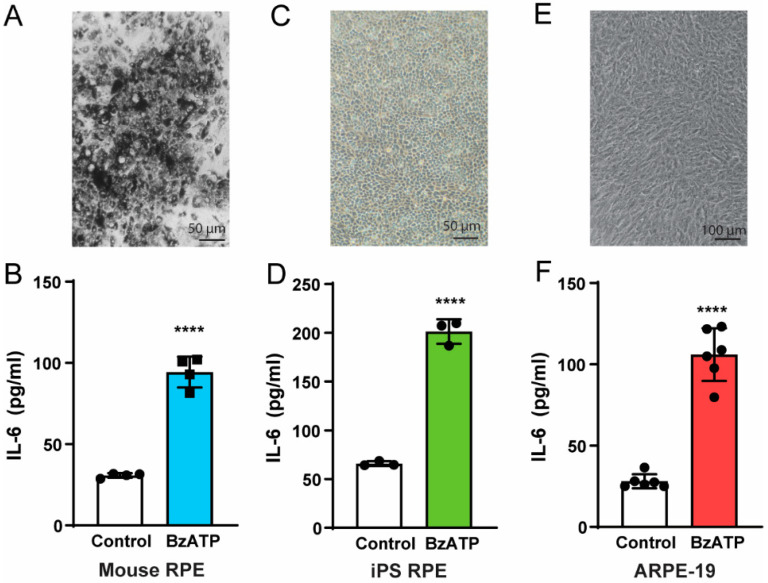
BzATP triggers IL-6 release from mouse, iPS-derived RPE cells and ARPE-19 cells. (**A**) Representative image of cultured mouse RPE cells, 11 days after plating. (**B**) Levels of IL-6 in the bath from primary cultures of mouse RPE cells increases when exposed to 50 µM BzATP for 1 h (**** *p* < 0.001, *n* = 4). (**C**) Image of iPS-derived RPE cells 8 days after plating. (**D**) iPS-RPE released IL-6 in response to BzATP (500 µM, 2 h; **** *p* < 0.001, *n* = 3). (**E**) ARPE-19 cells 10 days after plating. (**F**) ARPE-19 cells released IL-6 in response to BzATP (500 µM, 2 h; **** *p* < 0.001, *n* = 6).

Initial experiments asked whether stimulation of the P2X7 receptor with agonist BzATP triggered release of cytokine IL-6 from primary cultures of mouse RPE cells. Mouse RPE cells were grown until confluent (Figure 1A). Exposure of cells to 50 µM BzATP for 1 h increased the concentration of IL-6 in the bath over three-fold, from 31 pg/mL in control to 99 pg/mL in cells exposed to BzATP (Figure 1B). While these absolute levels are low, the minimum volume of solution needed to keep the cells moist had a height of ~3 mm, while the extracellular distance in vivo is submicron [25], suggesting cytokine concentration could be several orders of magnitude higher *in vivo*. BzATP also triggered a release of IL-6 from RPE cells derived from human iPS cells. Cells were grown for at least 2 weeks and had a cobblestone appearance (Figure 1C). BzATP exposure increased levels of IL-6 outside of iPS-RPE cells three-fold, from 66 pg/mL in control media to 201 pg/mL with BzATP exposure (Figure 1D).

Additional experiments were performed on the cultured human ARPE-19 cell line; cells were grown for 14 days with the last 10 days in 1% FBS to encourage a more mature phenotype [26] (Figure 1E). IL-6 levels were 28 pg/mL two h after addition of control medium, but rose to 106 pg/mL when BzATP (500 µM) was added to the medium (Figure 1F). The similarity in the responses from mouse RPE cells, iPS-RPE cells and ARPE-19 cells strongly suggests that release of IL-6 from RPE cells is a common response to stimulation of the P2X7 receptor.

### 3.2. Polarity of P2X7 Receptor Stimulation and IL-6 Release

**Figure 2:**
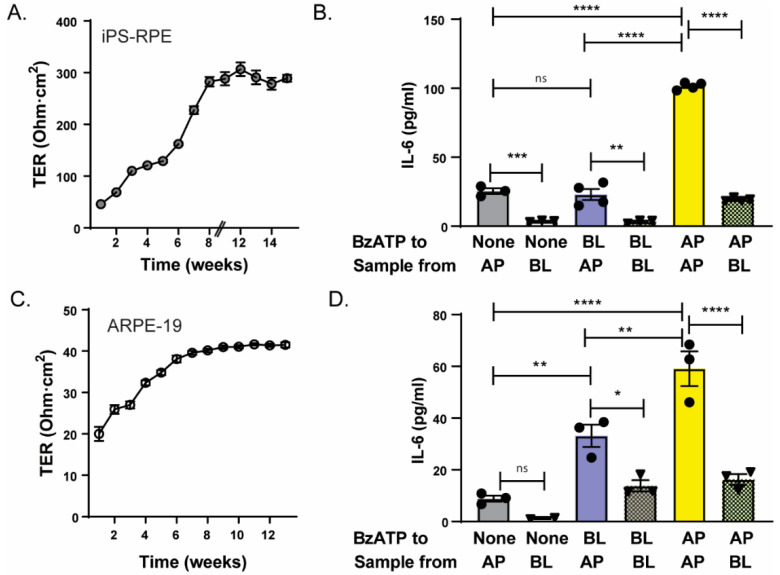
Polarity of IL-6 release. (**A**) Time-dependent changes in TEER of iPS-RPE cells grown on Transwell chambers. Symbols represent the mean ± SEM (*n* = 11). (**B**) Polarized release of IL-6 from iPS-RPE cells sampled from the apical (AP) or basolateral (BL) sides of the Transwell insert 2 h after 500 µM BzATP was added to the apical (AP), basolateral (BL) or neither (None) compartment. One way ANOVA F(5,15) = 308.0, *p* < 0.0001 with Tukey post hoc test (** *p* < 0.01, *** *p* < 0.001, **** *p* < 0.0001, ns = not significant; *n* = 3–4). (**C**) Time-dependent increases in TEER of ARPE-19 cells grown in Transwell inserts. Symbols represent the mean ± SEM (*n* = 20–30). (**D**) Polarized release of IL-6 from ARPE-19 cells sampled from the apical (AP) or basolateral (BL) sides of the Transwell chamber 2 h after 500 µM BzATP was added to the apical (AP), basolateral (BL) or neither (None) compartment. One way ANOVA F(5,11) = 30.56, *p* < 0.0001 with Tukey’s post-hoc test, * *p* < 0.05, ** *p* < 0.01, **** *p* < 0.0001, ns = not significant.

RPE cells form part of the blood retinal barrier, with their apical membrane facing the outer segments of retinal photoreceptors and their basolateral membrane adjacent to the choroidal blood supply [27]. While previous immunohistochemical staining of mouse retina suggested the P2X7 receptor was present on both apical and basolateral membranes [28], the polarity of IL-6 release to stimulation from either side was examined to focus on functional polarity. iPS-RPE cells were grown on permeable cell culture inserts for several months; the transepithelial electrical resistance (TEER) rose to 290 Ω·cm^2^ after 8 weeks (Figure 2A), consistent with the establishment of tight junctions and polarity [29]. Baseline levels of IL-6 in control solutions were low in chambers facing both apical and basolateralmembranes, although levels were >5-fold higher in samples taken from the apical chamber as compared to the basal chamber (Figure 2B). Addition of BzATP to the apical chamber increased levels of IL-6 in both chambers, but concentrations in the apical chamber were >5-fold greater than the basal chamber.

Polarity experiments were repeated in ARPE-19 cells grown on permeable cell culture inserts. TEER rose to 59 Ω·cm^2^ after 8 weeks and remained stable at this level (Figure 2C), consistent with levels previously reported [29], and significantly lower than TEER in iPS-RPE cells (*p* < 0.0001). In spite of the reduced TEER levels, however, the pattern of polarized IL-6 release from ARPE-19 cells was similar to that from iPS-RPE cells (Figure 2D). Addition of BzATP to the apical chamber led to a large IL-6 release, with levels in the apical chamber four-fold greater than the basal chamber. The main difference between the responses in the iPS-RPE and ARPE-19 cells was a general reduction in IL-6 concentration, and a greater response to basal application of BzATP; whether this reflects P2X7 receptors on the basolateral membrane of ARPE-19 cells or seepage of BzATP due to the lower TEER is unknown. Regardless, results from both iPS-RPE and ARPE-19 cells indicate that the primary release of IL-6 occurred across the apical membrane, with the greatest response found when BzATP was applied to the apical membrane.

### 3.3. Pharmacological Validation of the P2X7 Receptor

**Figure 3:**
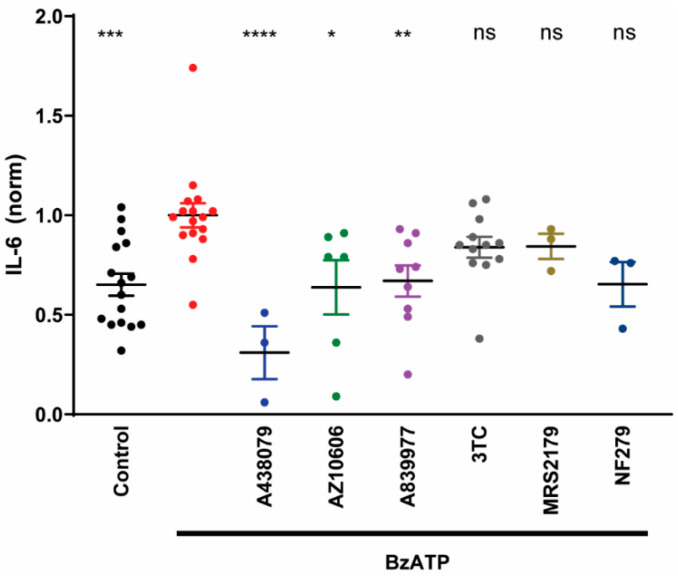
Pharmaceutical validation of P2X7 receptor. Exposure of ARPE-19 cells to 100 µM BzATP in isotonic solution for 30 min triggered a release of IL-6 (*** vs. Control, *p* = 0.0005, *n* = 13). In the presence of BzATP, IL-6 release was reduced by P2X7 antagonists A438079 (50 µM, **** *p* < 0.0001, *n* = 3), AZ10606120 (50 µM AZ10606, * *p* = 0.0113, *n* = 6) and A839977 (50 µM, ** *p* = 0.0071, *n* = 9). No significant reduction (ns) in IL-6 levels was found when lamivudine (3TC, 100 µM, *n* = 12), MRS2176 (300 nM, *n* = 3) or NF 279 (50 nM, *n* = 3) were added in the presence of BzATP. One way ANOVA (F97,60) = 5.42, *p* < 0.0001; Dunnett’s multiple comparisons test vs. BzATP alone.

The P2X7 receptor has been previously identified in RPE cells by multiple groups using immunohistochemistry, PCR, and functional analysis [28,30,31,32]. Although BzATP is widely used as an agonist for the P2X7 receptor, it can also interact with P2X1 and P2Y1 receptors [33,34,35,36]. To confirm that the actions of BzATP were mediated by the P2X7 receptor, multiple antagonists were tested for their ability to block the release of IL-6 from RPE cells in the presence of BzATP (Figure 3). Competitive P2X7 antagonist A438079 significantly blocked the release of IL-6 triggered by BzATP; A438079 had little or no activity at other P2X receptors [35]. The actions of BzATP were also blocked by AZ10606120, a negative allosteric modulator of the human P2X7 receptor [37], and by antagonist A839977 [38]; all three antagonists produced a near complete block of IL-6 release. Lamivudine (3TC) is used clinically as a nucleoside reverse transcriptase inhibitor but has also been shown to inhibit the actions of the P2X7 receptor in mice [39]. However, the reduction in BzATP-mediated IL-6 release by 3TC was not significant. Likewise, neither P2Y1 antagonist MRS2176 nor P2X1 antagonist NF 279 produced a significant reduction in IL-6 levels in the presence of BzATP. Taken together, these results suggest that the P2X7 receptor is primarily responsible for the BzATP-mediated release of IL-6 from RPE cells.

### 3.4. IL-6 Release through Rapid Rise in Ca^2+^

**Figure 4:**
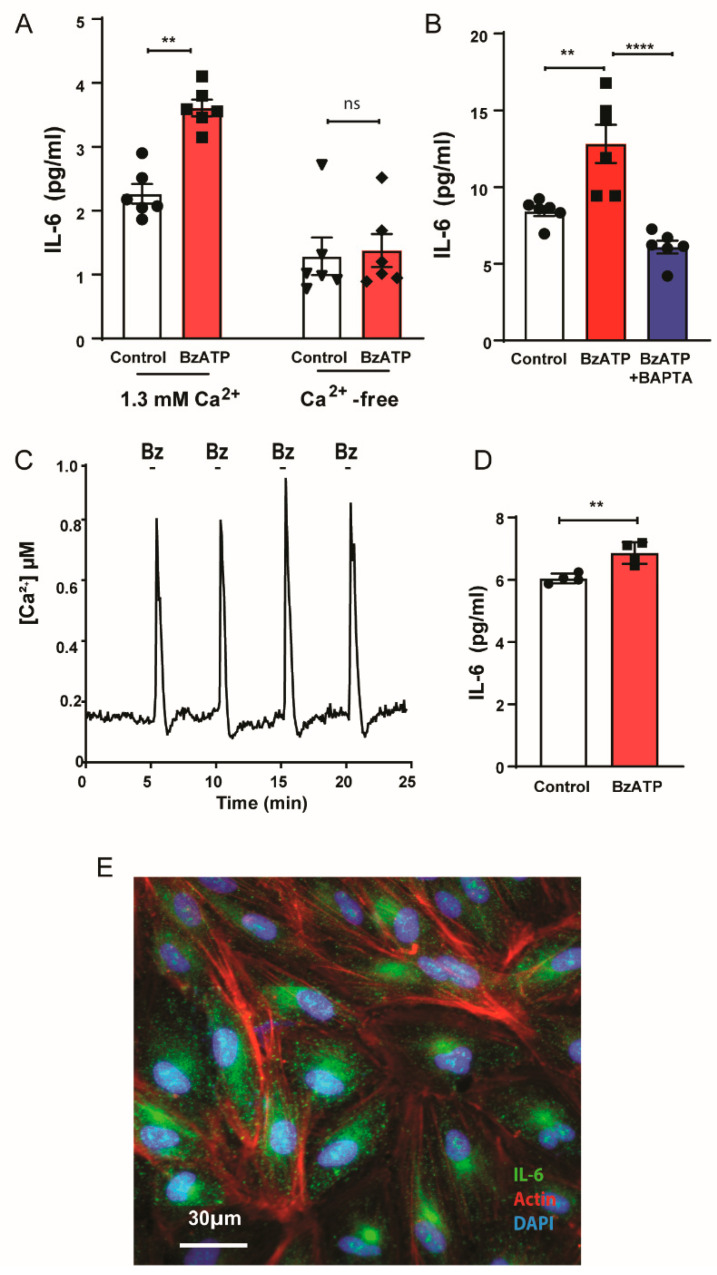
Calcium and IL-6 release from ARPE-19 cells. (**A**) The release of IL-6 triggered by BzATP (50 µM) was dependent upon extracellular Ca^2+^. (One-way ANOVA F(3,20) = 23.89 *p* < 0.0001; Tukey’s post hoc test; ** *p* = 0.0018, ns = not significant, *n* = 6). IL-6 levels were significantly increased after 15 min in Ca^2+^ containing isotonic solution with 1.3 mM Ca^2+^, but the rise in Ca^2+^ associated by BzATP was abolished in Ca^2+^-free solution. (**B**) Chelation of Ca^2+^ with 20 µM BAPTA-AM prevented the release of IL-6 after 30 min with BzATP (200 µM) One-way ANOVA F(2,15) = 19.17 *p* < 0.0001; Dunnet’s post hoc test **** *p* < 0.0001; ** *p* = 0.0018, *n* = 6). (**C**) BzATP (50 µM) led to a rapid rise in intracellular calcium when applied for 15 s that was reversible and repeatable. (**D**) Exposure to 50 µM BzATP for 1 min led to a small but significant IL-6 release (unpaired *t*-test ** *p* = 0.005, *n* = 4). Experiments in (**A**–**D**) were performed in the absence of Mg^2+^ to prevent its block of the P2X7 receptor. (**E**) Particulate staining for IL-6 in unstimulated cells; Actin (phaloidin red), DAPI (blue).

The P2X7 receptor is an ionotropic channel permeable to cations including Ca^2+^ [40], and the electrochemical gradients imply a substantial entry of Ca^2+^ into the RPE cells after receptor stimulation under physiological conditions. As Ca^2+^ is implicated in the release of numerous cytokines, the requirement for extracellular Ca^2+^ in the BzATP-mediated release of IL-6 was examined. While application of BzATP released IL-6 in the presence of control solution containing a physiologically relevant 1.3 mM extracellular Ca^2+^, the response was abolished when extracellular Ca^2+^ was removed (Figure 4A). To confirm a role for intracellular Ca^2+^ in the release of IL-6, cells were exposed to 20 µM cell permeable calcium chelator bis(2-aminophenoxy)ethane tetraacetic acid acetoxymethyl (BAPTA-AM); cells were preincubated for 1 h in BAPTA-AM, then exposed to BzATP in the presence of BAPTA-AM. BAPTA-AM prevented the rise of IL-6 in cells exposed to BzATP (Figure 4B).

The magnitude and time course of changes to cytoplasmic Ca^2+^ in response to BzATP application were determined. Cytoplasmic Ca^2+^ rose rapidly after application of BzATP, with robust, reversible, and repeatable spikes of Ca^2+^ found after application of BzATP for only 15 s (Figure 4C). To determine whether IL-6 release also showed a rapid response, cells were exposed to BzATP for only 1 min; this brief exposure led to a significant, albeit small, release of IL-6 (Figure 4D). Together, these rapid rises in cytoplasmic Ca^2+^ and extracellular IL-6 release suggested the influx of Ca^2+^ acted upon pre-formed stores of IL-6. Immunocytochemistry of baseline, unstimulated cells detected IL-6 in round clusters, consistent with vesicular staining of IL-6 (Figure 4E). Together, these observations are consistent with a mechanism in which the rapid elevation of cytoplasmic Ca^2+^ following stimulation of the P2X7 receptor leads to fusion of pre-formed vesicles containing IL-6 with the plasma membrane and release of IL-6 across this membrane.

### 3.5. P2X7 Receptor and IL-1β Release

**Figure 5:**
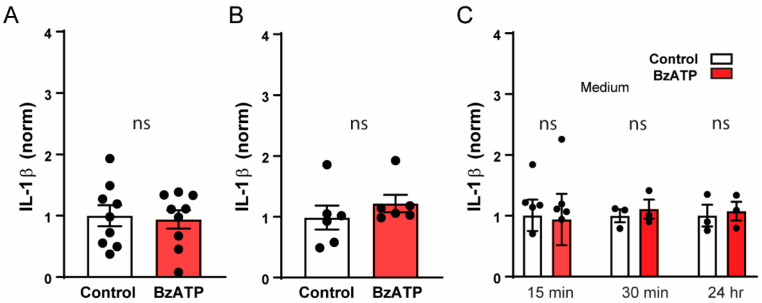
Stimulation of P2X7 receptor did not trigger release of IL-1β from unprimed ARPE-19 cells. The levels of IL-1β in the bath surrounding cells in control isotonic (white) and 50μM BzATP (red) solutions after 15 min (**A**; *n* = 9, *p* = 0.79) or 30 min (**B**; *n* = 6, *p* = 0.37). (**C**) Cells in growth media were incubated in BzATP for 15 min (*n* = 6), 30 min (*n* = 3) and 24 h (*n* = 3), but there was no rise in bath levels of IL-1β. One –way ANOVA F(5,18) = 0.033, *p* = 0.9993.

The master cytokine IL-1β has been reported to lead to a secondary upregulation and release of IL-6 [41]. In RPE cells, release of IL-6 was previously shown to be stimulated by exposure to IL-1β for 4 h [42]. Given that the P2X7 receptor is associated with assembly and activation of the NLRP3 inflammasome, which in turn leads to the maturation and release of IL-1β [6], we asked whether the release of IL-6 evoked by P2X7 receptor stimulation was due to IL-1β release. BzATP did not lead to the release of IL-1β under the unprimed conditions associated with IL-6 release. There was no difference in the levels of IL-1β in the bath 15 and 30 min after addition of BzATP when compared to levels in control isotonic solution (Figure 5A,B). To ensure the lack of response did not reflect the absence of a key substance in the environment, experiments were repeated in cell media. Cells were incubated in BzATP for 15 min, and 30 min in growth media, and to examine the effect of prolonged stimulation, cells were exposed to BzATP for 24 h, but again, BzATP had no effect on levels of IL-1β (Figure 5C). Of note, neither these cells, nor the ones showing IL-6 release in Figure 1, Figure 2, Figure 3 and Figure 4, were primed with lipopolysaccharide to prime the NLRP3 inflammasome. Together, these findings imply that the release of IL-6 triggered by P2X7 activation did not require IL-1β.

### 3.6. Enhanced IL-6 Release from ABCA4^−/−^ RPE Cells

**Figure 6:**
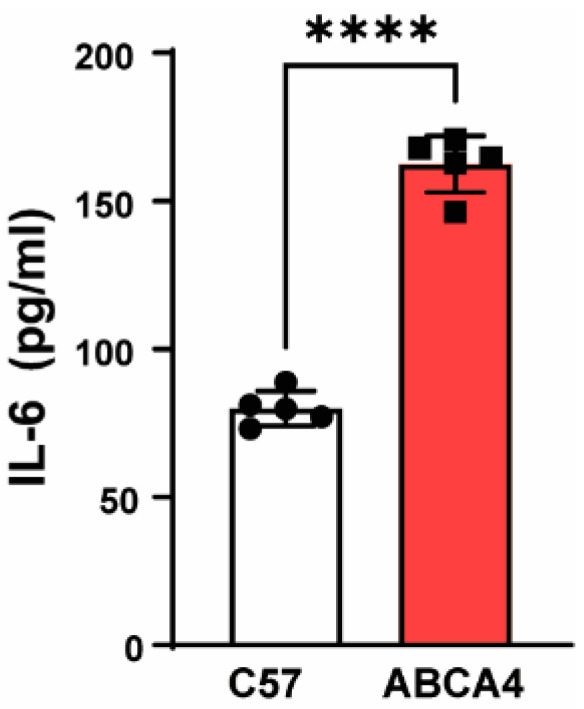
P2X7 preceptor-mediated IL-6 release increased in ABCA4^−/−^ RPE cells. Primary cultures of RPE cells from C57Bl6 and ABCA4^−/−^ mice were exposed to IL-6 for 1 h, after which the supernatant was collected and IL-6 levels determined. **** *p* < 0.0001, *n* = 5.

The ABCA4^−/−^ mouse is a model of recessive Stargardt’s retinopathy with known defects in both RPE and photoreceptor cells [43]. We have previously shown that expression of the P2X7 receptor is elevated in RPE cells from the ABCA4^−/−^ mouse [28]. As sampling in the subretinal space is difficult, we cultured RPE cells from ABCA4^−/−^ mice and control C57Bl6 mice to determine if IL-6 levels were indeed higher after stimulation of the P2X7 receptor. Exposure to BzATP was associated with increased levels of IL-6 bathing RPE cells from ABCA4^-/-^ mice as compared to wildtype controls (Figure 6).

## 4. Discussion

The primary conclusions from this study are that stimulation of the P2X7 receptor leads to the release of cytokine IL-6 from RPE cells. The release can be rapid, requires influx of Ca^2+^ from outside, and is independent of IL-1β. The detection of the IL-6 release in response to BzATP from primary cultures of mouse RPE cells, RPE cells derived from human iPS cells, and from the ARPE-19 cell line demonstrates the wide-spread nature of the response.

It is difficult to accurately sample IL-6 levels in vivo, given that the sub-retinal space separating RPE cells and photoreceptors is only 10–20 nm across [44], and the complex extracellular layers of Bruch’s membrane on the basolateral membrane preclude non-invasive sampling [45]. This difficulty is compounded by the nature of extracellular ATP signaling; intracellular levels of ATP are several orders of magnitude greater that extracellular concentrations, and ATP is widely released in response to mechanical stimulation. Probes that push against and rupture cells can themselves lead to release of ATP during attempts to obtain samples of extracellular ATP [46,47]. While measurements from cultured cells cannot completely recapitulate the in vivo situation, the identification of the P2X7 receptor on RPE cells in vivo using molecular, protein, and functional assays [28,32,48], combined with the use of three different cell models in the current study in Figure 1 helps overcome these limitations and supports the response across species and cell types. The detection of polarized IL-6 release in response to BzATP from iPS-RPE and ARPE-19 cells grown on permeable supports shown in Figure 2 adds to the relevance of this release.

### 4.1. Identification of P2X7 Receptors

ATP is the endogenous agonist for the P2X7 receptor [49], but its low sensitivity (pEC_50_ of 2.6 in mouse and 4.1 in human P2X7 receptors), combined with its ability to stimulate other P2X and P2Y receptors at lower concentrations, makes BzATP preferable as an experimental agonist [35,50]. BzATP activates the P2X7 receptor at lower concentrations than ATP (pEC_50_ of 4 and 5.3 at mouse and human P2X7 receptors, respectively). The demonstration that three different antagonists inhibit the ability of BzATP to trigger IL-6 release increased confidence for involvement of the P2X7 receptor in the current study. The specificity of antagonist A438079 for the P2X7 receptor makes involvement of other receptors unlikely [35]. A839977 is a tetrazole derivative with a high degree of specificity and selectivity for the P2X7 receptor [38,51]. The reduction of IL-6 release in the presence of negative allosteric modulator AZ10606120 further supports receptor identity [37]. Although BzATP can also act at P2X1 and P2Y1 receptors [33,34,36], neither P2X1 antagonist NF 279 nor P2Y1 antagonist MRS2176 significantly reduced the BzATP-mediated release of IL-6. Together, the data in Figure 3 strongly implicate the P2X7 receptor in the release of IL-6 triggered by BzATP.

### 4.2. Mechanism of Release P2X7 Receptor-Mediated IL-6 Release

The results presented in Figure 4 are consistent with IL-6 release following the influx of Ca^2+^ after stimulation of the P2X7 receptor. The P2X7 receptor is an ionotropic cation channel with substantial permeability to Ca^2+^; careful calculation of the relative permeability of the channel from the fractional contribution of Ca^2+^ to the total ATP-gated membrane current suggest the channel has *P*_Ca_/*P*_Na_ ≤ 1 [52]. The absence of IL-6 release in Ca^2+^-free solutions, combined with the lack of release in the presence of Ca^2+^ chelator BAPTA, the detection of IL-6 release within minutes, the rapid elevation of intracellular Ca^2+^ following application of BzATP, and the presence of IL-6 staining with vesicular patterning in unstimulated cells all support the hypothesis that Ca^2+^ entry rapidly triggers IL-6 release from preformed vesicles. The detailed mechanisms underlying the release of IL-6 triggered by the P2X7 receptor from RPE cells are unknown, however. Ca^2+^-dependent vesicular release is usually due to binding of Ca^2+^ to synaptotagmins and activation of SNARE machinery [53,54], and synaptotagmin 1 is constitutively expressed in RPE cells [55]. It was recently reported that, in astrocytes, stimulation of the P2X7 receptor led to IL-6 release after 5–6 h through involvement of NADPH oxidase and ROS production downstream of Ca^2+^ entry [56]. In microglial cells, 24 h exposure to BzATP increased expression of IL-6 mRNA and release of IL-6, while 4 h exposure to BzATP increased immunocytochemical staining for IL-6 [57]. The rapid release of preformed cytokines is usually associated with classic innate immune cells types such as eosinophils, mast cells, and neutrophils [53], so the presence of rapid Ca^2+^-dependent IL-6 release in RPE cells may be of interest.

While evidence for a rapid Ca^2+^-dependent release of IL-6 after P2X7 receptor stimulation is strong, elevation of IL-6 expression cannot be ruled out on a longer time scale. In isolated optic nerve head astrocytes, IL-6 expression was increased 4 h after exposure to BzATP, while intravitreal injection of BzATP increased retinal IL-6 expression after 24 h [17]. Expression of IL-6 can also be increased by IL-1β, with increased release detected after 4 h [42]. However, the rapid time course of IL-6 release in RPE cells, combined with data in Figure 5 showing the inability of BzATP to trigger IL-1β release under the same conditions capable of causing IL-6 release, imply that the IL-6 response is independent of IL-1β. This study does not negate the possibility that, under conditions where the NLRP3 inflammasome is “primed”, P2X7 receptor stimulation could release IL-1β and lead to a secondary upregulation and/or release of IL-6. Of relevance here is the recent report that P2X7 receptors can increase the priming of inflammasome genes in astrocytes in addition to its role in activation [58]. However, the present study indicates that IL-1β release is not necessary for IL-6 release by the P2X7 receptor in RPE cells.

### 4.3. Physiological Relevance

The physiological relevance of P2X7 receptor-mediated IL-6 release from RPE cells depends on the conditions leading to stimulation of the receptor in addition to the effects of IL-6. High concentrations of ATP are necessary for endogenous receptor activation [59]. Localized release of ATP from conduits such as pannexin channels can provide sufficient ATP for autocrine stimulation of adjacent P2X7 receptors [60]. Within the retina and RPE, pathways for regulating levels of ATP are tightly controlled [46,61,62]. ATP release from RPE cells occurs following stimulation of the NMDA receptor for glutamate [63], activation of the CFTR channel [64], and from the ATP store within lysosomes following stimulation of the TLR3 receptor [65]. Whether these pathways will generate sufficient ATP to stimulate the P2X7 receptor in the tight subretinal space in vivo is unknown. However, NTPDase1, a marker of extracellular ATP levels [66], was elevated in the RPE/choroid of the ABCA4^-/-^ mouse model of retinal degeneration [28], consistent with increased extracellular ATP levels under chronic diseased conditions. RPE cells from ABCA4^−/−^ mice also express increased levels of the P2X7 receptor [28], and data in Figure 6 indicate these cells release more IL-6 than control. Whether this contributes to the pathogenesis involved in Stargardt’s Disease remains to be determined.

The consequences of IL-6 release are expected to be complex; while IL-6 is frequently considered a “pro-inflammatory” cytokine, it can be both protective and pathological [15], with the presence of both membrane bound and soluble receptors for IL-6 influencing the outcome [67]. Classical signaling of IL-6 involves binding to the IL-6 receptor, leading to interaction of gp130 activation of Jak/Stat pathways and proinflammatory consequences. The trans-signaling pathway involves IL-6 first binding to a soluble IL-6 receptor, which in turn binds to membrane bound gp130, with trans-signaling associated with regenerative or anti-inflammatory signaling [68]. IL-6 was associated with photoreceptor protection in a model of retinal detachment, [69], and IL-6 can protect retinal ganglion cells [70,71]. However, IL-6 has recently been associated with epithelial to mesenchymal transition (EMT) [72,73], and type 2 EMT is recognized in the RPE pathology associated with AMD [74,75,76]. Whether increased IL-6 release following P2X7 receptor stimulation actually contributes to EMT in RPE cells is a subject of future investigation.

## 5. Conclusions

These data indicate that stimulation of the P2X7 receptor leads to the release of cytokine IL-6 from RPE cells through a process involving Ca^2+^ influx. This release of IL-6 may increase inflammatory or protective signaling in RPE cells under conditions where extracellular ATP levels rise.

## References

[1] Peeters P.M., Wouters E.F., Reynaert N.L. (2015). Immune homeostasis in epithelial cells: Evidence and role of inflammasome signaling reviewed. J. Immunol. Res..

[2] Glaser L., Coulter P.J., Shields M., Touzelet O., Power U.F., Broadbent L. (2019). Airway epithelial derived cytokines and chemokines and their role in the immune response to respiratory syncytial virus infection. Pathogens.

[3] Ferrari D., Pizzirani C., Adinolfi E., Lemoli R.M., Curti A., Idzko M., Panther E., Di Virgilio F. (2006). The P2X7 receptor: A key player in IL-1 processing and release. J. Immunol..

[4] Savio L.E.B., de Andrade Mello P., da Silva C.G., Coutinho-Silva R. (2018). The P2X7 receptor in inflammatory diseases: Angel or demon?. Front. Pharmacol..

[5] Dinarello C.A., Simon A., van der Meer J.W. (2012). Treating inflammation by blocking interleukin-1 in a broad spectrum of diseases. Nat. Rev. Drug Discov..

[6] Kelley N., Jeltema D., Duan Y., He Y. (2019). The NLRP3 inflammasome: An overview of mechanisms of activation and regulation. Int. J. Mol. Sci..

[7] Wu L., Zhou C., Wu J., Chen S., Tian Z., Du Q. (2020). Corticosterone inhibits LPS-induced NLRP3 inflammasome priming in macrophages by suppressing xanthine oxidase. Mediat. Inflamm..

[8] Lakkaraju A. (2012). Endo-lysosome function in the retinal pigment epithelium in health and disease. Adv. Exp. Med. Biol..

[9] Sparrow J.R., Hicks D., Hamel C.P. (2010). The retinal pigment epithelium in health and disease. Curr. Mol. Med..

[10] Detrick B., Hooks J.J., Klettner A.K., Dithmar S. (2020). The RPE cell and the immune system. Retinal Pigment Epithelium in Health and Disease.

[11] Tarallo V., Hirano Y., Gelfand B.D., Dridi S., Kerur N., Kim Y., Cho W.G., Kaneko H., Fowler B.J., Bogdanovich S. (2012). DICER1 loss and Alu RNA induce age-related macular degeneration via the NLRP3 inflammasome and MyD88. Cell.

[12] Doyle S.L., Ozaki E., Brennan K., Humphries M.M., Mulfaul K., Keaney J., Kenna P.F., Maminishkis A., Kiang A.S., Saunders S.P. (2014). IL-18 attenuates experimental choroidal neovascularization as a potential therapy for wet age-related macular degeneration. Sci. Transl. Med..

[13] Gao J., Cui J.Z., To E., Cao S., Matsubara J.A. (2018). Evidence for the activation of pyroptotic and apoptotic pathways in RPE cells associated with NLRP3 inflammasome in the rodent eye. J. Neuroinflamm..

[14] Tseng W.A., Thein T., Kinnunen K., Lashkari K., Gregory M.S., D’Amore P.A., Ksander B.R. (2013). NLRP3 inflammasome activation in retinal pigment epithelial cells by lysosomal destabilization: Implications for age-related macular degeneration. Investig. Ophthalmol. Vis. Sci..

[15] Spooren A., Kolmus K., Laureys G., Clinckers R., De Keyser J., Haegeman G., Gerlo S. (2011). Interleukin-6, a mental cytokine. Brain Res. Rev..

[16] Sato K., Takeda A., Hasegawa E., Jo Y.-J., Arima M., Oshima Y., Ryoji Y., Nakazawa T., Yuzawa M., Nakashizuka H. (2018). Interleukin-6 plays a crucial role in the development of subretinal fibrosis in a mouse model. Immunol. Med..

[17] Lu W., Albalawi F., Beckel J.M., Lim J.C., Laties A.M., Mitchell C.H. (2017). The P2X7 receptor links mechanical strain to cytokine IL-6 up-regulation and release in neurons and astrocytes. J. Neurochem..

[18] Guha S., Baltazar G.C., Tu L.A., Liu J., Lim J.C., Lu W., Argall A., Boesze-Battaglia K., Laties A.M., Mitchell C.H. (2012). Stimulation of the D5 dopamine receptor acidifies the lysosomal pH of retinal pigmented epithelial cells and decreases accumulation of autofluorescent photoreceptor debris. J. Neurochem..

[19] Maguire J.A., Gagne A.L., Jobaliya C.D., Gandre-Babbe S., Gadue P., French D.L. (2016). Generation of human control iPS cell line CHOPWT10 from healthy adult peripheral blood mononuclear cells. Stem Cell Res..

[20] Pashos E.E., Park Y., Wang X., Raghavan A., Yang W., Abbey D., Peters D.T., Arbelaez J., Hernandez M., Kuperwasser N. (2017). Large, diverse population cohorts of hiPSCs and derived hepatocyte-like cells reveal functional genetic variation at blood lipid-associated loci. Cell Stem Cell.

[21] Duong T.T., Vasireddy V., Ramachandran P., Herrera P.S., Leo L., Merkel C., Bennett J., Mills J.A. (2018). Use of induced pluripotent stem cell models to probe the pathogenesis of Choroideremia and to develop a potential treatment. Stem Cell Res..

[22] Lu W., Gómez N.M., Lim J.C., Guha S., Campagno K.E., McCaughey S.A., Laties A.M., Carlsson L.G., Mitchell C.H. (2018). The P2Y12 receptor antagonist ticagrelor reduces lysosomal pH and autofluorescence in retinal pigmented epithelial cells from the ABCA4-/- mouse model of retinal degeneration. Front. Pharmacol. Exp. Pharmacol. Drug Discov..

[23] Virginio C., Church D., North R.A., Surprenant A. (1997). Effects of divalent cations, protons and calmidazolium at the rat P2X7 receptor. Neuropharmacology.

[24] Gómez N.M., Lu W., Lim J.C., Kiselyov K., Campagno K.E., Grishchuk Y., Slaugenhaupt S.A., Pfeffer B.A., Fliesler S.J., Mitchell C.H. (2017). Robust lysosomal calcium signaling through channel TRPML1 is impaired by lysosomal lipid accumulation. FASEB J..

[25] Uehara F., Yasumura D., LaVail M.M. (1991). Rod- and cone-associated interphotoreceptor matrix in the rat retina. Differences in light-evoked distributional changes. Investig. Ophthalmol. Vis. Sci..

[26] Samuel W., Jaworski C., Postnikova O., Krishnan Kutty R., Duncan T., Tan L.X., Poliakov E., Lakkaraju A., Redmond T. (2017). Appropriately differentiated ARPE-19 cells regain phenotype and gene expression profiles similar to those of native RPE cells. Mol. Vis..

[27] Strauss O. (2005). The Retinal pigment epithelium in visual function. Physiol. Rev..

[28] Guha S., Baltazar G.C., Coffey E.E., Tu L.-A., Lim J.C., Beckel J.M., Eysteinsson T., Lu W., O’Brien-Jenkins A., Patel S. (2013). Lysosomal alkalinization, lipid oxidation, impaired autophagy and reduced phagosome clearance triggered by P2X7 receptor activation in retinal pigmented epithelial cells. FASEB J..

[29] Lehmann G.L., Benedicto I., Philp N.J., Rodriguez-Boulan E. (2014). Plasma membrane protein polarity and trafficking in RPE cells: Past, present and future. Exp. Eye Res..

[30] Dutot M., Liang H., Pauloin T., Brignole-Baudouin F., Baudouin C., Warnet J.M., Rat P. (2008). Effects of toxic cellular stresses and divalent cations on the human P2X7 cell death receptor. Mol. Vis..

[31] Yang D., Elner S.G., Clark A.J., Hughes B.A., Petty H.R., Elner V.M. (2011). Activation of P2X receptors induces apoptosis in human retinal pigment epithelium. Investig. Ophthalmol. Vis. Sci..

[32] Yang D. (2017). Targeting the P2X7 receptor in age-related macular degeneration. Vision.

[33] Vigne P., Hechler B., Gachet C., Breittmayer J.P., Frelin C. (1999). Benzoyl ATP is an antagonist of rat and human P2Y1 receptors and of platelet aggregation. Biochem. Biophys. Res. Commun..

[34] Allsopp R.C., El Ajouz S., Schmid R., Evans R.J. (2011). Cysteine scanning mutagenesis (residues Glu52-Gly96) of the human P2X1 receptor for ATP: Mapping agonist binding and channel gating. J. Biol. Chem..

[35] Syed N.-I.-H., Kennedy C. (2012). Pharmacology of P2X receptors. Wiley Interdiscip. Rev. Membr. Transp. Signal..

[36] Ohtomo K., Shatos M.A., Vrouvlianis J., Li D., Hodges R.R., Dartt D.A. (2011). Increase of intracellular Ca^2+^ by purinergic receptors in cultured rat lacrimal gland myoepithelial cells. Investig. Ophthalmol. Vis. Sci..

[37] Michel A.D., Chambers L.J., Walter D.S. (2008). Negative and positive allosteric modulators of the P2X(7) receptor. Br. J. Pharmacol..

[38] Honore P., Donnelly-Roberts D., Namovic M., Zhong C., Wade C., Chandran P., Zhu C., Carroll W., Perez-Medrano A., Iwakura Y. (2009). The antihyperalgesic activity of a selective P2X7 receptor antagonist, A-839977, is lost in IL-1alphabeta knockout mice. Behav. Brain Res..

[39] Mizutani T., Fowler B.J., Kim Y., Yasuma R., Krueger L.A., Gelfand B.D., Ambati J. (2015). Nucleoside reverse transcriptase inhibitors suppress laser-induced choroidal neovascularization in mice. Investig. Ophthalmol. Vis. Sci..

[40] Liang X., Samways D.S.K., Cox J., Egan T.M. (2019). Ca(^2+^) flux through splice variants of the ATP-gated ionotropic receptor P2X7 is regulated by its cytoplasmic N terminus. J. Biol. Chem..

[41] Cahill C.M., Rogers J.T. (2008). Interleukin (IL) 1beta induction of IL-6 is mediated by a novel phosphatidylinositol 3-kinase-dependent AKT/IkappaB kinase alpha pathway targeting activator protein-1. J. Biol. Chem..

[42] Elner V.M., Scales W., Elner S.G., Danforth J., Kunkel S.L., Strieter R.M. (1992). Interleukin-6 (IL-6) gene expression and secretion by cytokine-stimulated human retinal pigment epithelial cells. Exp. Eye Res..

[43] Weng J., Mata N.L., Azarian S.M., Tzekov R.T., Birch D.G., Travis G.H. (1999). Insights into the function of Rim protein in photoreceptors and etiology of Stargardt’s disease from the phenotype in abcr knockout mice. Cell.

[44] Steinberg R.H., Wood I. (1974). Pigment epithelial cell ensheathment of cone outer segments in the retina of the domestic cat. Proc. R Soc. Lond. B Biol. Sci..

[45] Curcio C.A., Johnson M., Ryan S.J., Sadda S.R., Hinton D.R., Schachat A.P., Sadda S.R., Wilkinson C.P., Wiedemann P., Schachat A.P. (2013). Chapter 20—Structure, Function, and Pathology of Bruch’s Membrane. Retina.

[46] Mitchell C.H., Reigada D. (2008). Purinergic signalling in the subretinal space: A role in the communication between the retina and the RPE. Purinergic Signal..

[47] Linden J., Koch-Nolte F., Dahl G. (2019). Purine release, metabolism, and signaling in the inflammatory response. Annu. Rev. Immunol..

[48] Kerur N., Hirano Y., Tarallo V., Fowler B.J., Bastos-Carvalho A., Yasuma T., Yasuma R., Kim Y., Hinton D.R., Kirschning C.J. (2013). TLR-independent and P2X7-dependent signaling mediate Alu RNA-induced NLRP3 inflammasome activation in geographic atrophy. Investig. Ophthalmol. Vis. Sci..

[49] Di Virgilio F., Giuliani A.L., Vultaggio-Poma V., Falzoni S., Sarti A.C. (2018). Non-nucleotide agonists triggering P2X7 receptor activation and pore formation. Front. Pharmacol..

[50] North R.A., Surprenant A. (2000). Pharmacology of cloned P2X receptors. Annu. Rev. Pharmacol. Toxicol..

[51] Donnelly-Roberts D.L., Namovic M.T., Han P., Jarvis M.F. (2009). Mammalian P2X7 receptor pharmacology: Comparison of recombinant mouse, rat and human P2X7 receptors. Br. J. Pharmacol..

[52] Liang X., Samways D.S., Wolf K., Bowles E.A., Richards J.P., Bruno J., Dutertre S., DiPaolo R.J., Egan T.M. (2015). Quantifying Ca^2+^ current and permeability in ATP-gated P2X7 receptors. J. Biol. Chem..

[53] Stanley A.C., Lacy P. (2010). Pathways for cytokine secretion. Physiology.

[54] Murray R.Z., Stow J.L. (2014). Cytokine secretion in macrophages: SNAREs, Rabs, and membrane trafficking. Front. Immunol..

[55] Uhl P.B., Szober C.M., Amann B., Alge-Priglinger C., Ueffing M., Hauck S.M., Deeg C.A. (2014). In situ cell surface proteomics reveals differentially expressed membrane proteins in retinal pigment epithelial cells during autoimmune uveitis. J. Proteom..

[56] Munoz F.M., Patel P.A., Gao X., Mei Y., Xia J., Gilels S., Hu H. (2020). Reactive oxygen species play a role in P2X7 receptor-mediated IL-6 production in spinal astrocytes. Purinergic Signal..

[57] Shieh C.H., Heinrich A., Serchov T., van Calker D., Biber K. (2014). P2X7-dependent, but differentially regulated release of IL-6, CCL2, and TNF-α in cultured mouse microglia. Glia.

[58] Albalawi F., Lu W., Beckel J.M., Lim J.C., McCaughey S.A., Mitchell C.H. (2017). The P2X7 receptor primes IL-1β and the NLRP3 inflammasome in astrocytes exposed to mechanical strain. Front. Cell Neurosci..

[59] Khakh B.S., Burnstock G., Kennedy C., King B.F., North R.A., Seguela P., Voigt M., Humphrey P.P. (2001). International union of pharmacology. XXIV. Current status of the nomenclature and properties of P2X receptors and their subunits. Pharmacol. Rev..

[60] Dahl G. (2015). ATP release through pannexon channels. Philos. Trans. R Soc. Lond. B Biol. Sci..

[61] Sanderson J., Dartt D.A., Trinkaus-Randall V., Pintor J., Civan M.M., Delamere N.A., Fletcher E.L., Salt T.E., Grosche A., Mitchell C.H. (2014). Purines in the eye: Recent evidence for the physiological and pathological role of purines in the RPE, retinal neurons, astrocytes, Muller cells, lens, trabecular meshwork, cornea and lacrimal gland. Exp. Eye Res..

[62] Ventura A.L.M., dos Santos-Rodrigues A., Mitchell C.H., Faillace M.P. (2019). Purinergic signaling in the retina: From development to disease. Brain Res. Bull..

[63] Reigada D., Lu W., Mitchell C.H. (2006). Glutamate acts at NMDA receptors on fresh bovine and on cultured human retinal pigment epithelial cells to trigger release of ATP. J. Physiol..

[64] Reigada D., Mitchell C.H. (2005). Release of ATP from retinal pigment epithelial cells involves both CFTR and vesicular transport. Am. J. Physiol. Cell Physiol..

[65] Beckel J.M., Gomez N.M., Lu W., Campagno K.E., Nabet B., Albalawi F., Lim J.C., Boesze-Battaglia K., Mitchell C.H. (2018). Stimulation of TLR3 triggers release of lysosomal ATP in astrocytes and epithelial cells that requires TRPML1 channels. Sci. Rep..

[66] Lu W., Reigada D., Sevigny J., Mitchell C.H. (2007). Stimulation of the P2Y1 receptor up-regulates nucleoside-triphosphate diphosphohydrolase-1 in human retinal pigment epithelial cells. J. Pharmacol. Exp. Ther..

[67] Rose-John S. (2018). Interleukin-6 Family Cytokines. Cold Spring Harb. Perspect Biol..

[68] Rose-John S. (2012). IL-6 trans-signaling via the soluble IL-6 receptor: Importance for the pro-inflammatory activities of IL-6. Int. J. Biol. Sci..

[69] Chong D.Y., Boehlke C.S., Zheng Q.D., Zhang L., Han Y., Zacks D.N. (2008). Interleukin-6 as a photoreceptor neuroprotectant in an experimental model of retinal detachment. Investig. Ophthalmol. Vis. Sci..

[70] Echevarria F.D., Formichella C.R., Sappington R.M. (2017). Interleukin-6 deficiency attenuates retinal ganglion cell axonopathy and glaucoma-related vision loss. Front. Neurosci..

[71] Echevarria F.D., Rickman A.E., Sappington R.M. (2016). Interleukin-6: A constitutive modulator of glycoprotein 130, neuroinflammatory and cell survival signaling in retina. J. Clin. Cell Immunol..

[72] Zhou J., Zhang C., Pan J., Chen L., Qi S.T. (2017). Interleukin6 induces an epithelialmesenchymal transition phenotype in human adamantinomatous craniopharyngioma cells and promotes tumor cell migration. Mol. Med. Rep..

[73] Ebbing E.A., van der Zalm A.P., Steins A., Creemers A., Hermsen S., Rentenaar R., Klein M., Waasdorp C., Hooijer G.K.J., Meijer S.L. (2019). Stromal-derived interleukin 6 drives epithelial-to-mesenchymal transition and therapy resistance in esophageal adenocarcinoma. Proc. Natl. Acad. Sci. USA.

[74] Kimura K., Orita T., Liu Y., Yang Y., Tokuda K., Kurakazu T., Noda T., Yanai R., Morishige N., Takeda A. (2015). Attenuation of EMT in RPE cells and subretinal fibrosis by an RAR-γ agonist. J. Mol. Med..

[75] Radeke M.J., Radeke C.M., Shih Y.H., Hu J., Bok D., Johnson L.V., Coffey P.J. (2015). Restoration of mesenchymal retinal pigmented epithelial cells by TGFbeta pathway inhibitors: Implications for age-related macular degeneration. Genome Med..

[76] Ghosh S., Shang P., Terasaki H., Stepicheva N., Hose S., Yazdankhah M., Weiss J., Sakamoto T., Bhutto I.A., Xia S. (2018). A Role for betaA3/A1-Crystallin in Type 2 EMT of RPE Cells Occurring in Dry Age-Related Macular Degeneration. Investig. Ophthalmol. Vis. Sci..

